# One health surveillance: linking human and animal rabies surveillance data in Kenya

**DOI:** 10.3389/fpubh.2025.1594162

**Published:** 2025-06-16

**Authors:** Samuel Kahariri, Lian F. Thomas, Bernard Bett, Marianne Mureithi, Brian Njuguna, Nyamai Mutono, Thumbi Mwangi

**Affiliations:** ^1^Directorate of Livestock Policy, Research and Regulations, Nairobi, Kenya; ^2^International Livestock Research Institute, Nairobi, Kenya; ^3^Centre for Epidemiological Modelling and Analysis, University of Nairobi, Nairobi, Kenya; ^4^Faculty of Health Sciences, Department of Medical Microbiology and Immunology, University of Nairobi, Nairobi, Kenya; ^5^Royal (Dick) School of Veterinary Studies, University of Edinburgh, Midlothian, United Kingdom; ^6^Paul G. Allen School for Global Health, Washington State University, Pullman, WA, United States; ^7^Feed the Future Innovation Lab for Animal Health, Washington State University, Pullman, WA, United States; ^8^Institute of Immunology and Infection Research, University of Edinburgh, Edinburg, United Kingdom

**Keywords:** rabies, surveillance, one health, correlation, Bayesian, zoonotic

## Abstract

**Introduction:**

Rabies poses a significant public health and economic challenge in Kenya. The Kenya rabies elimination strategy identifies surveillance as a key pillar to achieve the targets of ending human deaths from rabies by 2030. Here we investigated the utility of the national human and animal rabies surveillance data to provide robust surveillance data to guide the Kenya rabies elimination program.

**Methods:**

We conducted a retrospective analysis of the official rabies data obtained from the national human and animal health surveillance systems between 2017 and 2023. We obtained data on bites, cases, and deaths in dogs and humans due to rabies. We estimated incidences and tested the relationships between rabies variables in human and dogs as a proxy for robust data availability.

**Results:**

On average, there were 162 cases and 84 deaths in dogs, while in humans, there were 53 cases and 6 deaths. We found positive correlations between dog bites and cases of dog rabies [RR = 1.33, 95% credible interval (CI): 1.16, 1.54], deaths and rabies cases in dogs (RR = 1.09, 95% CI: 1.05, 1.14) and death cases and dog bites (RR = 1.46, 95% CI: 1.06, 1.98). However, relationships between rabies cases and dog bites in humans were not statistically significant (RR = 1.00, 95% CI: 0.98, 1.03), whereas rabies cases in dogs and humans were negatively correlated (RR = 0.82, 95% CI: 0.68, 0.94).

**Discussion:**

The findings indicate that Kenya’s rabies surveillance system effectively captures trends in dog rabies but has gaps in human rabies case reporting. The weak relationship between rabies cases and dog bites in humans and the negative correlation between rabies cases in dogs and humans point to potential underreporting of human cases, that could be possibly driven by misdiagnosis or limited access to healthcare, or effective post-exposure treatment.

**Conclusion:**

Understanding these relationships is critical for improving the surveillance systems that can effectively support the rabies elimination program.

## 1 Introduction

An estimated 75% of emerging pathogens are zoonotic in nature (1). In 2023, zoonotic diseases accounted for approximately 21.5 million disability-adjusted life years (DALYs), significantly contributing to global morbidity and mortality (2). Efforts to mitigate zoonotic threats have increasingly focused on early detection, improved surveillance, and coordinated public health interventions following a One Health approach (3).

Rabies is the top-prioritized One Health zoonotic disease in Sub-Saharan Africa, driven by its high fatality rate, epidemic potential, socio-economic impact, and the availability of effective interventions (4). The Stepwise Approach towards Rabies Elimination (SARE) was jointly developed by the Food and Agriculture Organization (FAO), the Global Alliance for Rabies Control (GARC), and the World Health Organization (WHO) as a practical tool for One Health planning, monitoring, and evaluation to support rabies-endemic countries like Kenya in developing and implementing sustainable rabies elimination strategies.

Despite the existence of effective vaccines for both humans and animals, the disease still exists in two-thirds of the countries worldwide (5). Annually, it accounts for approximately 59,000 human deaths, with the burden predominantly affecting underserved populations in Africa and Asia, where domestic dog vaccination coverage is often suboptimal and human rabies exposure is common (6, 7).

In Kenya rabies is among the top five prioritized zoonotic diseases (8). However, it remains endemic, with an estimated 2,000 human deaths annually, primarily due to dog bites (9). The disease poses a significant public health concern, particularly in rural areas of Kenya where access to post-exposure prophylaxis (PEP) is limited and stray dog populations remain high (10, 11). The common practice where owned dogs are left unconfined, increases the number of free-roaming and perceived stray dogs across the country (12). While effective rabies vaccines and immunoglobulins exist, their use remains suboptimal due to cost barriers and accessibility challenges (6, 7, 13).

Rabies control initiatives in Kenya are guided by the Strategic Plan for the Elimination of Human Rabies in Kenya 2014–2030 (SPEHRK) and are planned in accordance with SARE for Kenya to progress from its current endemic status to disease free status (11, 14). However, weaknesses in both human and animal surveillance systems, along with inadequate collaborations between the sectors, have been identified as major hinderances to effective rabies control in the country (11, 15). The strategy further highlights the role of effective integrated surveillance in enhancing early detection, timely responses and supporting monitoring of the impact of the control efforts. It advocates for strengthening collaboration between animal and public health sectors active stakeholder engagement in surveillance, establishment of an outbreak response plan and strengthening rabies diagnostic capacity in the country (3, 11).

Despite the critical role played by the surveillance system in rabies control, human rabies cases in Kenya are often underreported due to limited access to healthcare and diagnostic facilities, particularly in rural areas (16–18). The lack of integrated surveillance hampers the ability to track the disease’s transmission dynamics and implement timely control measures. Inadequate awareness among the human and animal health workers in disease surveillance tools and case definitions for priority diseases is a major cause for inconsistencies in reporting and therefore hampers the effectiveness of surveillance systems (19, 20). A standard case definition is an agreed set of criteria used to label an individual animal of human as having a condition of interest or not. The ministry of health in collaborations with World Health Organization (WHO) developed standard case definitions which included suspect and confirmed rabies cases (19). On the other hand, the animal diseases surveillance manual highlighted the key symptoms for rabies in animals but did not classify suspected cases from confirmed cases (21).

A previous study conducted in the Philippines utilized reported rabies cases, bites, and deaths in both humans and animals to describe the current rabies surveillance data and investigate the correlation between human and animal rabies. The study reported that early detection of rabies indicators in both humans and dogs has the potential to foster intersectoral collaborations between animal and public health sectors (22). In addition, the study demonstrated the potential utilization of rabies early warning indicators from animals and human passive surveillance system to set up a robust rabies early warning system and enhance integrated rabies surveillance that support rabies prevention and response, while also offering a scalable model for setting up an integrated system for other zoonotic diseases.

This study aimed to evaluate the relationship between human and animal rabies surveillance systems in Kenya by assessing the relationship between reported dog bites, rabies cases and rabies related deaths reported to the national surveillance systems in both sectors.

## 2 Methodology

**Figure 1:**
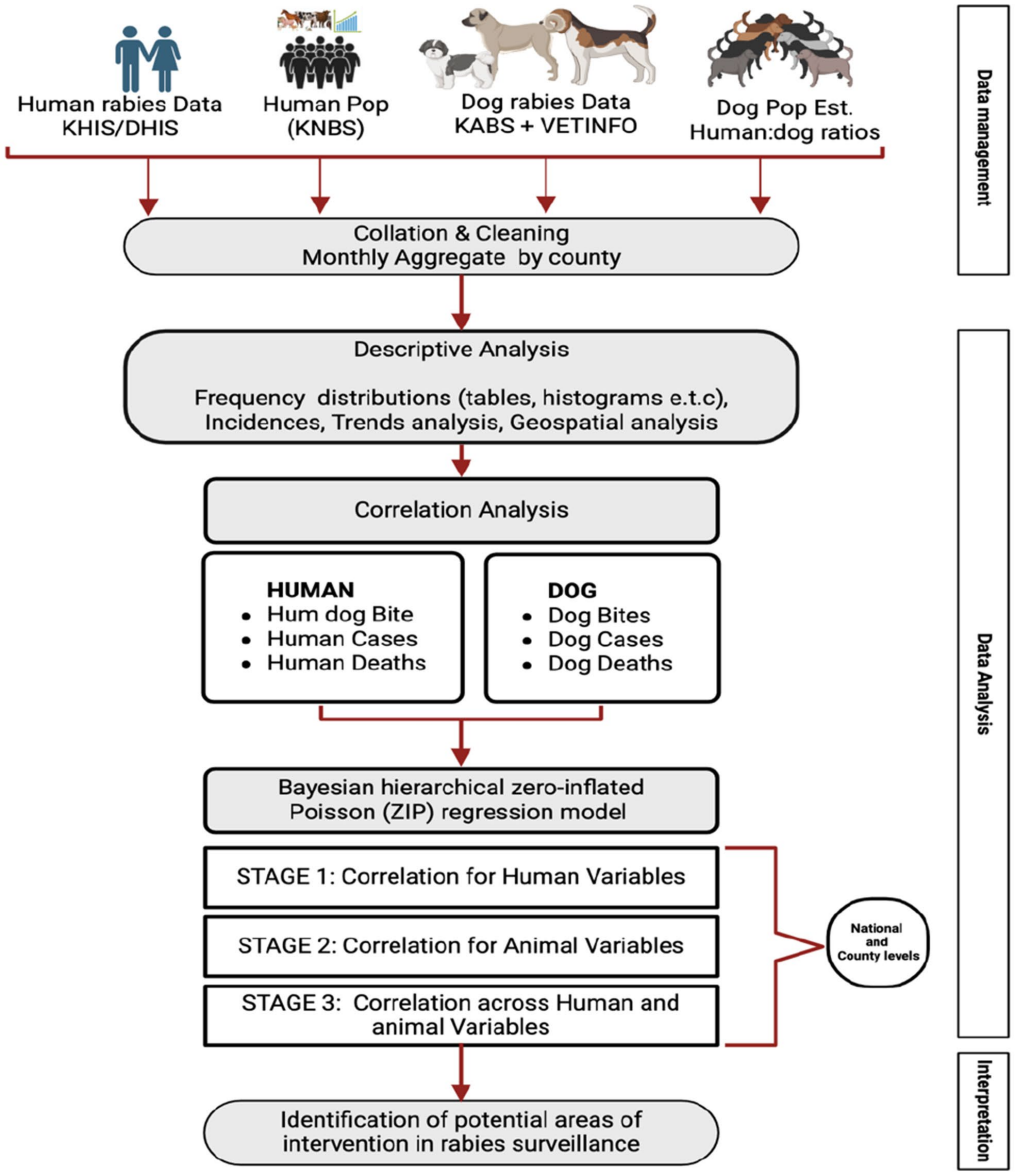
Summary of the official sources of human and animal rabies surveillance data, and the methodological approach used to determine the utility of these datasets for analysis that can guide the national rabies elimination program.

We conducted a retrospective analysis of human and dog rabies data obtained from the official surveillance systems for animals and humans for the period 2017 to 2023. The data obtained included reported dog bites, reported clinical cases of rabies and reported rabies related deaths. These data were used to perform descriptive and regression analyses (Figure 1).

### 2.1 Data source

We obtained records on both laboratory and clinically diagnosed cases of rabies in both human and livestock from the respective national surveillance systems; the Kenya Health Information System (KHIS) which collects data on human rabies cases from health facilities; and the Kenya Animal Biosurveillance System (KABS) that collects data on dog rabies from the frontline animal health workers around the country. In addition to KABS, we included data obtained from VETINFO google group which is a google online platform where data from regional laboratories in Kenya were channeled (23).

The collated retrospective data for all the variables of interest, as tabulated in the Table 1 below, were analyzed as monthly aggregates. The variables from human health surveillance were collected as aggregates at facility level while those in animal health surveillance were collected at individual animal level by the human health professionals and the animal health professional, respectively. Among all the variables, only human rabies cases have a case definition and a zero-reporting mechanism.

**Table 1:** Description of the variables under study.

Surveillance system	Variable name	Description of the variable	Source data
Human Health surveillance system	Human rabies cases	Clinical and laboratory cases of rabies in human	DHIS
	Human dog bites	Cases of bites to human by dogs	DHIS
	Human deaths	Deaths of human reported to arise from rabies	DHIS
Animal Health surveillance system	Dog rabies cases	Laboratory and clinically diagnosed cases of rabies in livestock	KABS, VETINFO
	Dog animal bites	Reported cases of bites in dogs. Also includes bites from other animals.	KABS, VETINFO
	Animal deaths	Deaths of livestock arising from rabies	KABS, VETINFO

**Table 2:** Tabulation of the stages of correlation analysis and the relationships investigated.

Stage	Correlations Models
Correlation of rabies parameters in animal health surveillance system	Dog rabies cases and dog animal bites
	Dog deaths and dog rabies cases
	Dog deaths and dog animal bites
Correlation of rabies parameters in human health surveillance system	Human dog bites and human rabies cases
	Human deaths and human rabies cases
	Human deaths and human dog bites
Correlation of rabies parameters in human and animal health surveillance system	Human rabies cases and dog rabies cases
	Human rabies cases and dog animal bites
	Human dog bites and dog rabies cases
	Human dog bites and dog animal bites

Yearly human population data disaggregated at a county level was obtained from the Kenya National Bureau of Statistics (KNBS) for the years 2017 to 2023. This data was used to calculate the incident rates for all the human-related variables.

**Figure 2:**
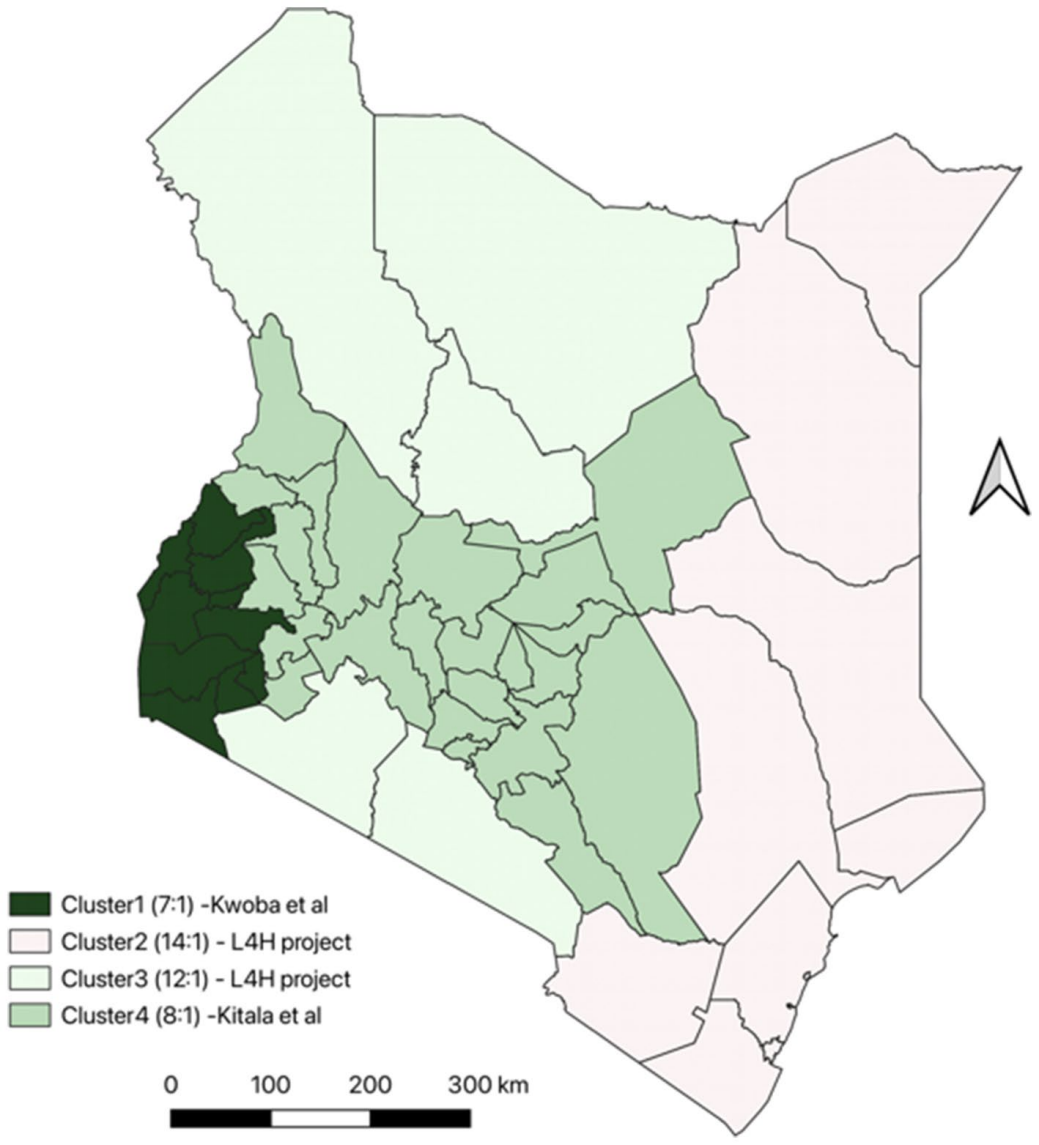
Dog population estimates in Kenya using previous ecological studies and project estimates.

Dog population estimates were calculated using the estimated human-to-dog population ratios derived from previous dog ecological studies and other project estimates in the country as shown in the Figure 2 below.

To calculate the dog population estimates, we divided the country into the following clusters based on the dog ecological studies undertaken previously, expert opinion and other available information on dog populations in Kenya;

1. Cluster 1 which included Kakamega, Vihiga, Bungoma, Busia, Siaya, Kisumu, Homa Bay, Migori, Kisii and Nyamira. The estimates for dog population was calculated using a Human to Dog population ratio of 7:1 was used to calculate based on ecological study in western Kenya (24).
2. Cluster 2 which included Garissa, Wajir, Mandera, Kilifi, Kwale, Lamu, Mombasa, Taita Taveta and Tana River. These were calculated using an estimated human-to-dog ratio of 14:1. This is because the counties mainly practice Islamic religion where keeping of dogs is not a common practice.
3. Cluster 3 included Marsabit, Turkana, Samburu, Narok, Kajiado. These were calculated using an estimated human-to-dog ratio of 12:1 which was calculated using data from a cluster randomized trial conducted in Marsabit (Livestock for Health) (25). These areas are mostly pastoralist and use dogs for grazing and security for livestock. (L4H).
4. Cluster 4 included all the other counties not in the 3 regions above, the Human: dog population ratio used to estimate the dog population was 8:1 based on a dog ecological study conducted in Machakos. We assumed that the factors influencing the dog ownership in this area were like Machakos county (15).

The formula used for estimating the dog population is as shown below. The dog population estimate was obtained by dividing the human population for the specifies counties by the identified human dog population ratio.

### 2.2 Data analysis

All data were cleaned and analyzed using R version 4.1.1 (26) and QGIS 3.16.7.

We undertook spatial analysis to determine the spatial distribution for all the rabies variables in human and animal surveillance systems. This was achieved through plotting the annual incidences per a million population per county and species. The incidence for rabies cases per a million population was computed using the formula below


$$\mathrm{Incidence}=\frac{Total\;\;casesforthewholeyear}{populationinthatyear}*1,000,000$$

We employed a hierarchical zero-inflated Poisson (ZIP) mixed effect model within the Bayesian framework to assess the correlations between variables in the dataset. The ZIP model accounted for the excess zeros that were not explained by the standard Poisson process (unstructured zeros). To capture spatial variability, we included a random slope to capture the varying relationships between the independent variables and the response across counties as well as a random intercept to account for differences in the number of cases between counties. We implemented the model using R-INLA package (27). Our findings were compared against previously documented household and herd-based studies.

We applied the R-INLA default prior distributions for the global intercept $\left(\;\beta_{0}\right)$ , fixed effects ( $\beta_{i}$ ) and the random intercepts ( $u_{i}$ ). For $u_{i},$ a gamma distribution was applied on the precision parameter $\tau =\frac{1}{\sigma^{2}}$ , specifically $\tau ∼Gamma\left(1,5\times 10^{-5}\right)$ and $\beta_{0}∼N\left(0,\tau =0\right)$ , $\beta_{i}∼N\left(0,\tau =10^{-3}\right).$

**Figure 3:**
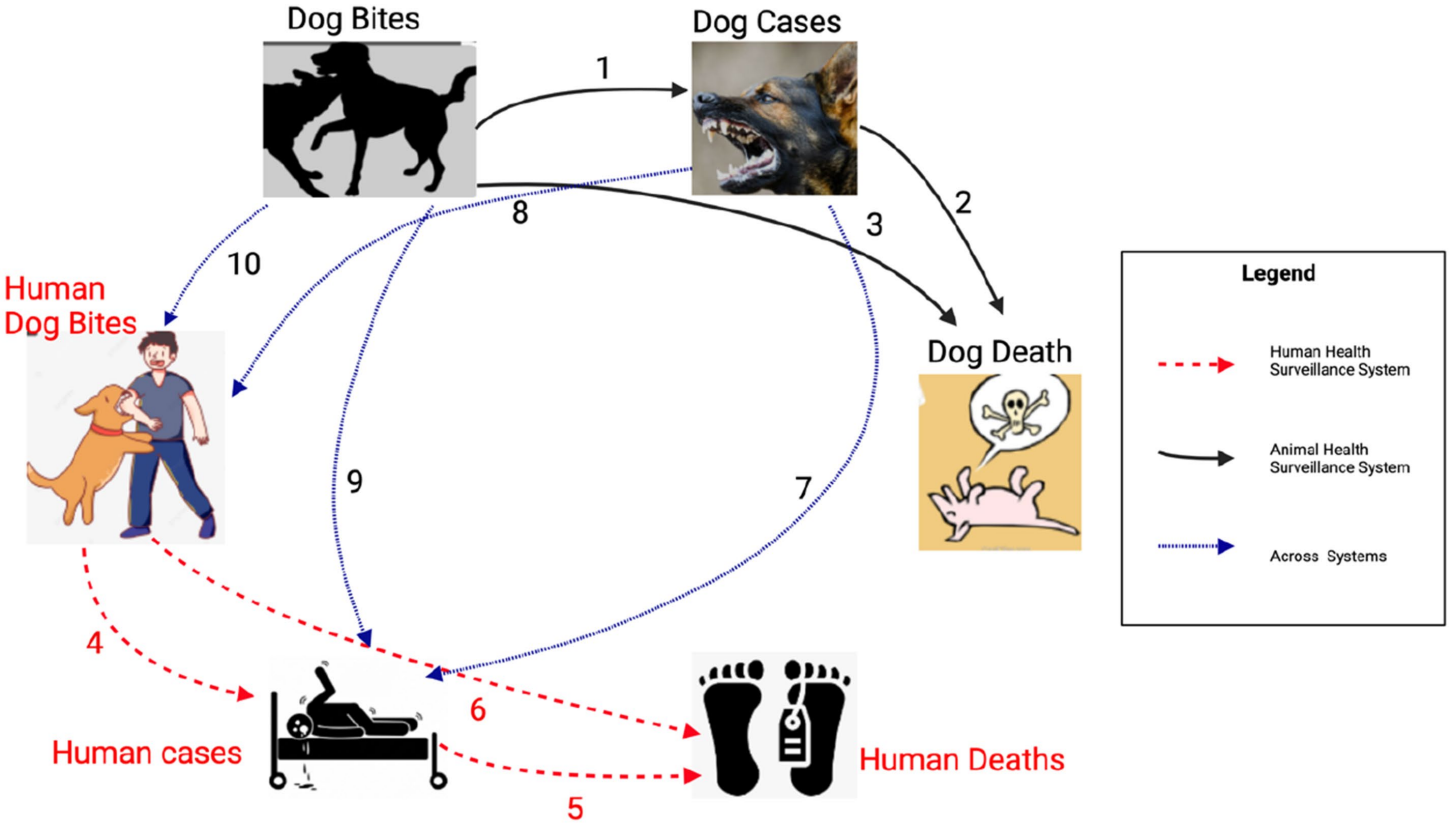
Illustration of the stages followed in the correlation analysis.

As shown in the Table 2 above Figure 3 below, the first stage tested correlations amongst the variables within the animal health surveillance system, followed by the correlations among variables within the human health surveillance system and finally we tested the correlation of variables across the human and animal health surveillance systems. Lastly, we assessed the effectiveness of the rabies control interventions in the identified counties as per the Strategic Plan for the Elimination of Human Rabies in Kenya 2014–2030 (SPEHRK). The below outlines the correlation models that were fitted per stage.

We drew 1,000 samples from the posterior distribution, to summarize both fixed and random effects. The county-specific intercept was obtained by adding the global intercept to the corresponding county-specific random intercept. Similarly, the county-specific slope was derived by summing the global slope (fixed effect) with the county-specific random slope. To assess the significance of random effects, we examined the credible intervals of their variance. Fixed effects were presented as Relative Risks (RRs) to express the relationship between dog bites and rabies cases.

The limitation of this study is the lack of official population estimates for dogs and use of retrospective surveillance data which may be subject to reporting bias and missing information. However, as the study aimed to assess the existing surveillance system, we utilized the available data to identify key gaps and inform necessary improvements. Availability of data on the interventions that may have been undertaken during the study period like dog vaccinations and use of PEP was limited. However, triangulation of the results was undertaken using the counties with known efforts on the interventions

## 3 Results

The descriptive statistics (for the period 2017–2023) highlight the burden of rabies at the national level based on the official reports. On average, there were 3 dog bites, 7 rabies cases and 4 deaths in dogs per month. In humans, the monthly average was 53 rabies cases and 6 deaths.

**Figure 4:**
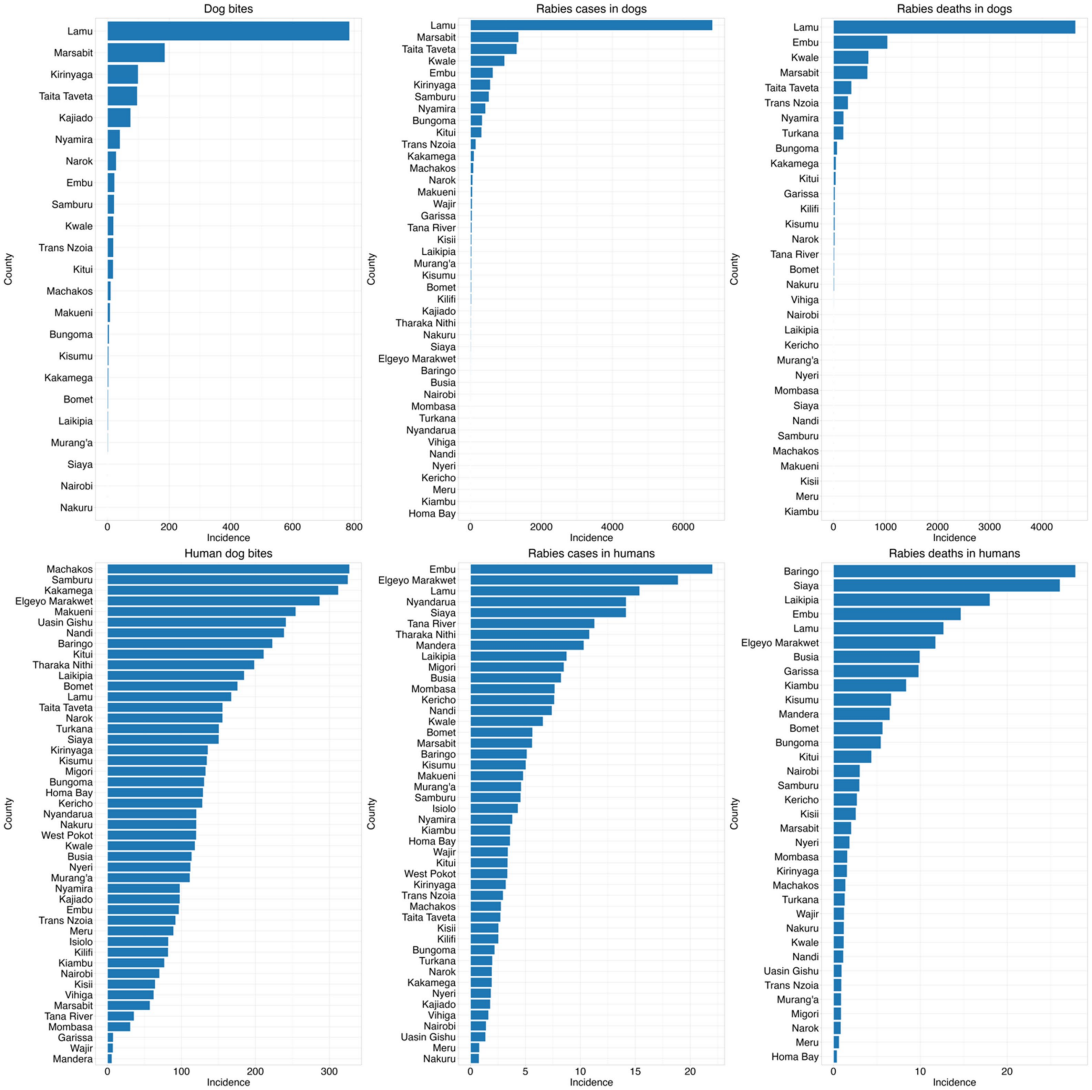
Incidences of bites, cases, and deaths in both human and animal at county level from 2017 to 2023 per million population.

Dog bites in humans were widespread, with an average of 6,417 bites per month as shown in the Table 3 below. With substantial variability as shown in Figure 4.

**Table 3:** Descriptive statistics of dog bites, cases and death (Counts and incidences per million population) for the period between 2017 to 2023 at national level (84 months).

Variable	Mean (Raw)	Min (Raw)	Max (Raw)	SD (Raw)	Total (Raw)	Mean (Inc)	Min (Inc)	Max (Inc)	SD (Inc)
Animal bites in dogs	3.19	0	25	4.72	268	0.54	0	4.23	0.79
Rabies cases in dogs	6.80	0	53	7.85	571	1.16	0	8.76	1.29
Rabies deaths in dogs	3.79	0	60	7.01	318	0.66	0	10.64	1.24
Dog bites in humans	6416.83	3,319	36,475	4151.4	539,014	131.96	69.75	743.45	85.8
Rabies cases in humans	53.43	7	337	44.6	4,488	1.11	0.14	7.09	0.94
Rabies deaths in humans	5.71	0	111	13.73	480	0.12	0	2.34	0.29

**Figure 5:**
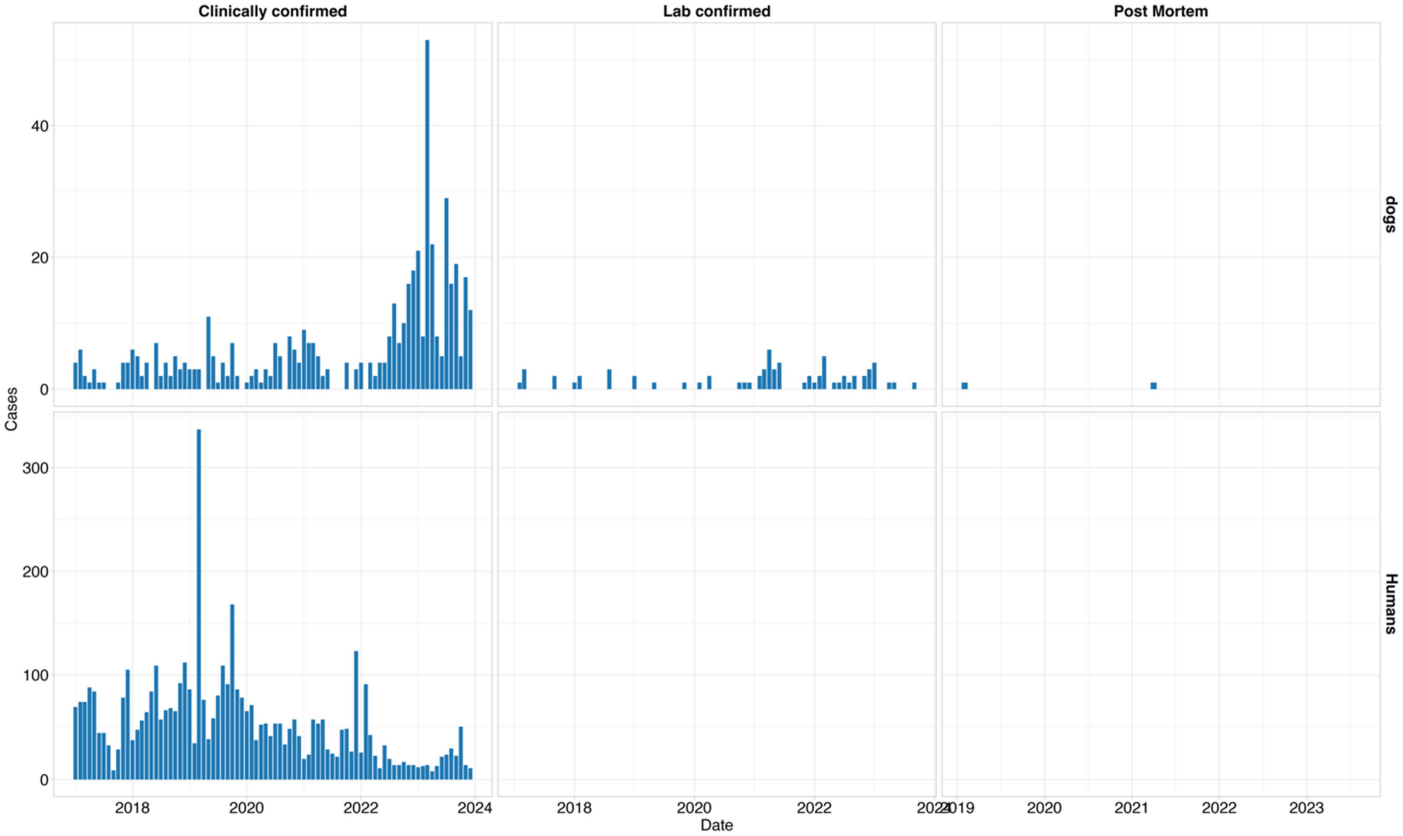
Frequencies of different modes of rabies diagnosis in humans and dogs from 2017 to 2023.

In dogs, the surveillance system captured most of the cases clinically (*n* = 499, 87%), followed by a few cases of laboratory confirmation (*n* = 70, 12%) and isolated reports using postmortem diagnosis (*n* = 2, 1%). In humans, all the reported cases were diagnosed clinically with no laboratory and postmortem diagnosis as shown in Figure 5 below. The highest number of clinically reported cases of human rabies was observed in 2019, which exceeded 300 in a single month, whereas the peak in reported dog rabies cases was in 2023 (Figure 5).

**Figure 6:**
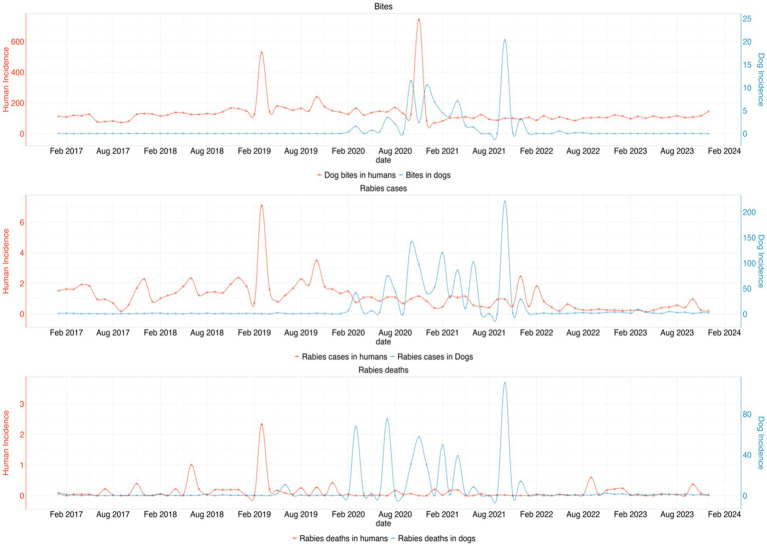
Trend of the incidences for dog bites, rabies cases, and rabies deaths in dogs and humans per million population from 2017 to 2023. The top graph shows a consistent pattern of dog bites in humans, with several peaks, particularly in early 2019 and 2020, while bites in dogs remain relatively low. The second graph highlights a marked increase in rabies cases in dogs during 2020 and 2021, with corresponding but lower peaks in human rabies deaths. The third graph shows strong peaks in rabies deaths in dogs with smaller but corresponding increases in human fatalities. However, a declining trend is observed from 2022 onwards across all variables.

From the incidence data, we observed several peaks of dog bite cases in humans in early 2019 and 2020, while reported bites in dogs remain relatively low. There was a marked increase in rabies cases in dogs during 2020 and 2021, with corresponding but lower peaks in human rabies deaths and strong peaks in rabies deaths in dogs with smaller but corresponding increases in human fatalities. In all variables, there was a declining trend from 2022 as shown in Figure 6 below.

The Figure 4 below illustrates the frequencies of the study variables in different counties. The county-level comparison of dog bites, rabies cases, and rabies deaths in both dogs and humans reveal distinct geographic patterns of rabies burden in Kenya. Notably, Lamu, Marsabit, Embu and Taita Taveta showed the highest incidences of rabies cases and deaths in dogs, whereas rabies cases in humans were predominantly found in Embu, Elgeyo Marakwet, Lamu and Nyandarua. A striking contrast was observed in counties like Kwale, Taita Taveta and Marsabit, where dog rabies cases and deaths were high, but human rabies cases remained relatively low. Conversely, in counties like Baringo, Samburu, and Elgeyo Marakwet, human rabies deaths were disproportionately high compared to dog rabies incidences.

**Figure 7:**
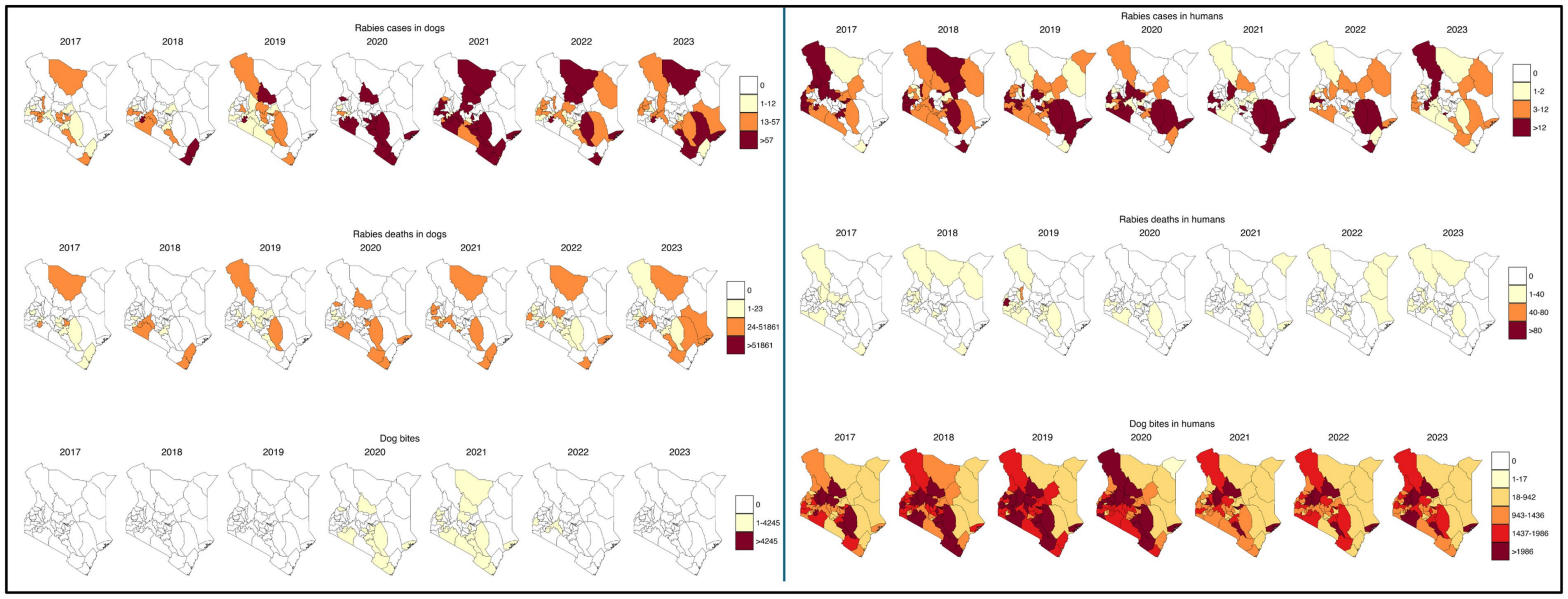
Spatial distribution of rabies variables (cases, dog bites and deaths) incidences per million population in humans and dogs from 2017 to 2023.

From the map in the Figure 7 below, rabies cases and deaths in dogs were concentrated in specific counties in central and northern region, with pronounced increases in areas like Lamu, Marsabit, and Kajiado from 2020 onward. In contrast, rabies cases and deaths in humans showed a broader distribution, with high incidences observed in counties like Embu, Baringo, Nyamira and Elgeyo Marakwet. Dog bites in humans were widespread, particularly in high-density areas. Human rabies deaths also showed substantial variation, with spikes in counties such as Baringo and Siaya, without corresponding spikes in human or dog cases of rabies suggesting significant underreporting or delayed interventions.

### 3.1 Correlation models at the national level

The results of our Bayesian hierarchical models examining the relationship between reported dog bites and dog cases, estimated a mean Relative Risk (RR) of 1.33 [95% Credible interval (CI): 1.16, 1.54]. For the dog cases & dog deaths model, (RR 1.09, 95% CI: 1.05, 1.14), indicating a significant relationship, similarly a significant association was observed between dog bites and dog deaths, with an RR of 1.46 (95% CI: 1.06, 1.98).

No association was found between dog bites in humans and human rabies cases (RR 1.00, 95% CI: 0.99, 1.00), Human rabies cases were positively and significantly associated with human rabies deaths (RR 1.05, 95% CI: 1.02, 1.10), while dog bites in humans had a significant negative correlation with human rabies deaths (RR: 0.997, 95% CI: 0.994, 0.999).

**Figure 8:**
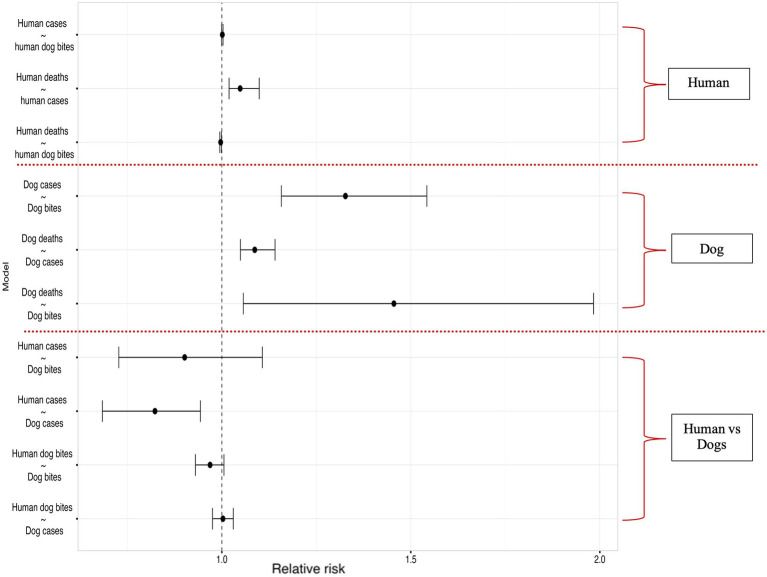
Results of the Bayesian hierarchical models examining correlations within the human rabies variables (between human rabies cases and human dog bites, human deaths and human rabies cases, human deaths and human dog bites), dog rabies variables (between dog cases and dog bites, dog deaths and dog cases, dog deaths and dog bites) and correlations across human and dog rabies variables (Dog bites and human rabies cases, dog rabies cases and human rabies cases, dog bites and human dog bites, human dog bites and dog rabies cases).

There was a significant negative correlation between dog rabies cases and human rabies cases, RR of 0.82 (95% CI: 0.68, 0.94) and no association found between dog bites in humans and dog rabies cases (RR: 1.00, 95% CI: 0.98, 1.03) or animal bites in dog and dog bites in humans (RR: 0.97, 95% CI: 0.93, 1.01, respectively). The Figure 8 below summarizes the correlation model results.

### 3.2 Correlation models at the county level

#### 3.2.1 Correlations between variables in individual surveillance systems

Correlation within human health surveillance systems at the county level revealed few significant associations across all relationships tested as shown in the supplementary material.

In animal health surveillance systems, results for the models ran between various sets of relationships between dog rabies parameters indicate that in majority of the counties, animal bites in dogs were positively correlated with cases of rabies in dogs. Animal bites in dogs were positively correlated with rabies associated deaths in dogs in almost all counties except in Turkana, and Embu counties. There were several cases where the tested relationships were not statistically significant owing to the poor data quality and under reporting. The details are shown in the supplementary materials.

#### 3.2.2 Correlation across human and animal surveillance systems

**Figure 9:**
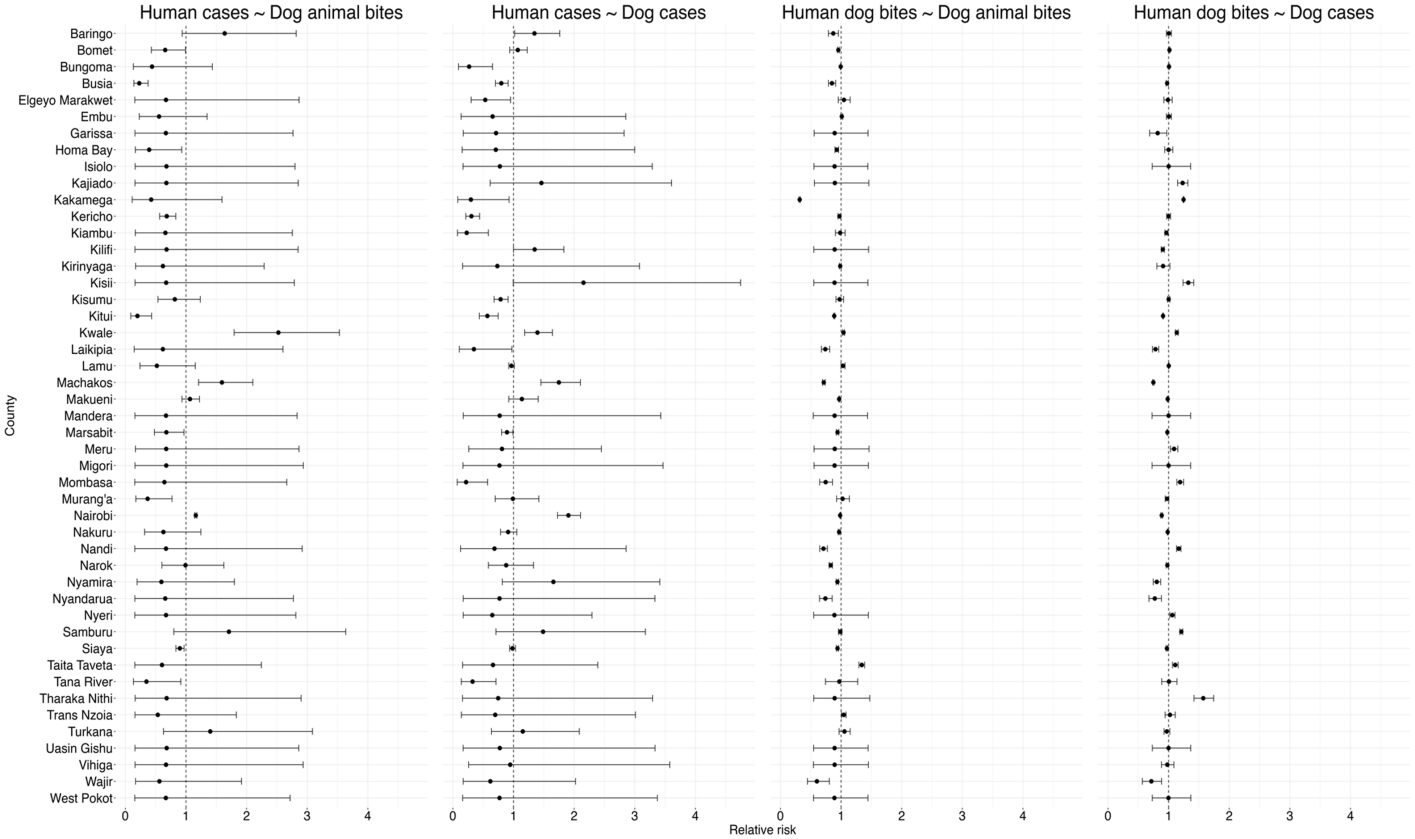
correlation between human and dog rabies related variables.

Generally, as shown in the Figure 9 below, the results revealed significant heterogeneity in the relationships between human and animal rabies surveillance data across counties in Kenya.

##### 3.2.2.1 Human cases and dog animal bites

Human rabies cases and dog bites was positively correlated in Nairobi, Machakos, and Kwale. The results show that the human dog bites are negatively correlated in Siaya, Tana River, Muranga, Marsabit, Kericho, Busia, Bomet, and Kitui counties while in the rest of the counties the variables were not significantly correlated as shown in Figure 9 below.

##### 3.2.2.2 Human cases and dog cases

Positive correlations were observed between human rabies cases and animal bites in dogs in several counties, suggesting a direct association between exposure and disease occurrence. Counties such as Nairobi, Machakos, Kwale, Kisumu, Baringo, Kilifi, demonstrate higher RR, suggesting a stronger positive correlation between rabies incidences in dogs and subsequent cases in humans. Conversely, several counties, including Tana River, Mombasa, Siaya, Marsabit, Kitui, Kisumu, Kiambu, Kericho, Busia and Bungoma RRs below 1, indicating a negative correlation between rabies cases in dogs and humans.

##### 3.2.2.3 Human dog bites and dog bites

Bites in dogs were seen to be positively correlated with human dog bites in Trans Nzoia, Taita Taveta, Lamu, and Kwale counties while it was negatively correlated in Wajir, Siaya, Nyamira, Narok, Nandi, Nakuru, Nairobi, Mombasa, Marsabit, Makueni, Machakos, Laikipia, Kitui, Homa Bay, Busia, Baringo, and Bomet counties as shown in the Figure 9 below.

##### 3.2.2.4 Human dog bites and dog cases

The Figure 9 below shows that there exists a positive correlation between dog rabies cases and human dog bites in Tharaka Nithi, Taita Taveta, Samburu, Nyeri, Nandi, Mombasa, Meru, Kwale, Kisii, Kakamega and Kajiado counties while a negative correlation was recorded in Siaya, Nyandarua, Nyamira, Narok, Nakuru, Nairobi, Makueni, Machakos, Laikipia, Kitui, Kilifi, Kiambu Garissa and Busia counties. In the rest of the counties, the correlation between the dog cases was not significantly correlated with human dog bites.

## 4 Discussion

The Kenya rabies elimination strategy has adopted a Stepwise Approach, emphasizing a multi-sectoral collaboration under the One Health framework, with surveillance and diagnostics being critical pillars. However, as confirmed in previous studies, our study highlights fragmented human and animal surveillance systems, and inadequate diagnostics, leading to poor case detection, increased human exposure, and preventable fatalities (28). Most rabies cases in both humans and animals were clinically diagnosed, with limited laboratory confirmation in animals and none for human cases within the surveillance system. This finding aligns with global trends, where most canine rabies cases are diagnosed clinically rather than through laboratory confirmation, despite the risk of misdiagnosis with other neurological conditions (6, 29). The lack of reliable laboratory-confirmed data further increases underreporting, making it difficult to accurately assess the burden of canine rabies and implement targeted interventions (6, 30).

This study analyzed Kenya’s data on dog bites, rabies cases and deaths in both humans and dogs to assess the relationship between the two surveillance systems. We observed that on average, 53 human rabies cases, six human rabies deaths and 6,417 human dog bite cases were reported per month. While dog rabies cases peaked in 2023, the highest number of human rabies cases occurred in 2019, indicating complex transmission dynamics. Regional disparities were evident, with Lamu, Marsabit, Embu, and Taita Taveta recording the highest burden of dog rabies cases, while Embu, Elgeyo Marakwet, Lamu, and Nyandarua reported the greatest number of human rabies cases. The correlation between dog rabies and human rabies incidence was weak, suggesting underreporting, diagnostic limitations, and surveillance gaps. We identified a strong association between dog rabies cases and dog deaths but an inconsistent relationship between human dog bites and human rabies cases, highlighting challenges in data reliability and surveillance sensitivity. A negative correlation between dog and human rabies cases further suggested gaps in timely case detection and intervention effectiveness.

The dog rabies surveillance data further indicate significant variability and inconsistencies, with high standard deviations in reported cases, bites, and deaths, suggesting heterogeneous reporting practices and possible underreporting. However, the analysis of the reporting rates is compromised by lack of a clear zero-reporting mechanism for rabies in animals which further complicates the identification of rabies free areas. This aligns with previous research indicating that passive surveillance often leads to rabies underreporting, particularly in low-resource settings (21). The over-reliance on clinical diagnosis for human rabies remains a major limitation, as atypical or early-stage symptoms may be misinterpreted (26, 27). The absence of postmortem diagnosis for human rabies cases may be linked to logistical or cultural barriers in obtaining tissue samples, as well as gaps in data sharing between pathology departments and national health surveillance systems (26). This emphasizes on the need to have clear standard case definitions and adequate awareness on the same among the human and animal health workers.

From our findings, we believe there exists regional disparities in rabies surveillance, with some areas demonstrating more effective monitoring while others remain inadequately covered as demonstrated by the different reporting rates and heterogeneity of the results across the counties. The increase in reported dog rabies cases from 2020 onward may indicate localized outbreaks detected by the surveillance system, or alternatively, may reflect enhanced surveillance and reporting in specific areas. Further, the strong correlation between increased dog bite incidents and rising rabies cases supports the role of dog bites as a key driver of rabies transmission within canine populations. These findings reinforce the need for targeted interventions, such as mass dog vaccination campaigns, to reduce transmission and bite-related exposures. This strategy mirrors successful fox rabies elimination programs in Europe, where targeted vaccination was implemented to eliminate the disease (31) and Mexico’s successful rabies elimination through 70% canine vaccination coverage (32).

Although not all dog bites result in rabies, as demonstrated in previous studies (9), the high rate of dog bites in humans, coupled with the relatively low number of reported rabies cases and deaths suggests either under-reporting of rabies cases or increased access to post-exposure prophylaxis (PEP), or misclassification of rabies cases as dog bites (7). Reports indicate that many health facilities around the country erroneously record clinical rabies cases as dog bites, contributing to significant under-reporting of human rabies cases, (Dr. Athman, MOH Per. Comms). This is due to limited awareness on the standard case defination among the health workers. The marked variability in human rabies cases and deaths across sub-national regions highlights potential inequities in access to rabies related interventions like vaccinations and PEP, particularly in resource-limited and remote regions (16, 20). The lower number of recorded deaths relative to cases further suggests under-reporting of rabies mortality. As demonstrated in prior research, inadequate knowledge of bite wound categorization and rabies case management among healthcare workers may contribute to misdiagnosis and low case detection within routine surveillance systems (20).

In human health surveillance system, rabies is classified as weekly reportable IDSR priority disease and has a case definition that classifies individuals with a dog bite or scratch as suspect cases of rabies (19). On the contrary, most of the reports received during the study period were more on human dog bites than rabies cases. This confirms inadequate awareness on the case definition and need to revise to align with the existing reporting tools. In the animal health surveillance, there exists no standardized case definition to guide the surveillance officers. These challenges compromises the quality of data and weakens the surveillance systems which contributes to lack of comprehensive data which was identified as a key barrier to rabies control (33).

The observed regional variability in rabies surveillance, differences in reporting practices, and the effectiveness of control measures align with findings that emphasize the role of veterinary staffing and surveillance funding as key determinants of rabies control success (29, 30). Studies in Nigeria and Cameroon have demonstrated that reinforcing surveillance networks and providing training for healthcare professionals significantly improves rabies exposure reporting and case detection (29, 30). These findings underscores the importance of sufficient staffing, financial investment, and continuous capacity building in achieving effective rabies surveillance and control. Additionally, the declining trend in rabies cases and deaths post-2021 may suggest improved control efforts, though further monitoring is necessary to demonstrate this. The peaks in dog bites and human rabies deaths in 2019 and 2020 point to potential outbreaks during this period, possibly increased by COVID-19-related disruptions to rabies control programs. Previous studies have shown that only 5% of African countries maintained planned rabies control activities during the pandemic (31), highlighting the vulnerability of elimination efforts to health system shocks.

To effectively introduce a One Health approach for rabies control, it is important to implement cross-sectoral coordination, integrated surveillance, and community engagement (34). Strengthening rabies surveillance systems by integrating human, animal, and environmental health data can improve outbreak detection and response (35). Studies from Bangladesh and Kenya highlight that the lack of intersectoral collaboration results in inefficient post-exposure prophylaxis (PEP) distribution and frequent vaccine stockouts, disproportionately affecting rural populations (20, 36). A digital One Health surveillance system, as implemented in Haiti, has proven to enhance real-time monitoring and optimize response strategies (37). Additionally, implementing community-led rabies education and bite management programs, like those in the Philippines, can reduce human exposure and ensure timely treatment access (38).

To achieve rabies elimination, there is a need to enhance rabies surveillance through innovations like rapid diagnostic tests and the adoption of integrated electronic case management, which improves case detection and response timeliness, particularly in resource-limited settings (35, 37). In resource limited countries like Kenya, tests like direct Rapid Immunohistochemical Test (dRIT) are more reliable and cost-effective for detection of rabies (39). Initiatives such as capacity building for health professionals, aimed at improving the clinical suspicion index among healthcare workers for the appropriate capturing and management of dog bites, should be prioritized (20). However, the full potential of these advancements is contingent upon robust inter-sectoral collaboration under a One Health framework and sustained investment to ensure equitable access to these technologies (35).

## 5 Conclusion

Despite Kenya’s ambition to eliminate human dog-mediated rabies by 2030, significant gaps in both human and canine surveillance systems such as underreporting, misreporting, weak diagnostic capacity, and inconsistent case definitions hinder accurate burden assessment and targeted interventions. These gaps stem from unclear data collection tools, low awareness among surveillance officers, the presence of an unsuitable case definition in the human health surveillance system, the absence of a case definition in the dog surveillance system, and the absence of an effective zero-reporting system for rabies cases and related variables. This further complicates the differentiation between actual underreporting and the true absence of cases.

To achieve rabies elimination, the heterogeneity and fragmented nature of surveillance efforts across counties suggest that rabies elimination strategies should be tailored to region-specific situations. The study identifies key interventions to drive rabies elimination, including enhancing rabies surveillance through the development and utilizing integrated electronic case management to improve case detection and response timeliness, particularly in resource-limited settings (35, 37). Kenya should adopt sustainable and cost-effective elimination strategies like vaccinating at least 70% of the domestic dogs coupled with laboratory-based animal rabies surveillance system and use of cost-effective diagnostic tests like dRIT (20, 39, 40)

The sustainable elimination of rabies also calls for a multifaceted approach that combines public awareness and education on wound care after dog bites, targeted training of healthcare professionals in WHO-recommended post-exposure prophylaxis (PEP) and clinical suspicion and case management (20). Finally, social mobilization and active community engagement are essential to foster a One Health approach, ensuring the effectiveness and sustainability of rabies surveillance and control efforts (35).

Without aggressive, targeted action driven by reliable data, Kenya risks losing ground in the fight against this deadly disease.

## Data Availability

The data analyzed in this study is subject to the following licenses/restrictions: The datasets used in this work were authorized by the responsible authorities and the GitHub link to the data can be availed upon request to the corresponding author. Requests to access these datasets should be directed to drkahariri@gmail.com.

## References

[1] Strengthening Global Health Security at the Human-Animal Interface. (2025). Available online at: https://www.who.int/activities/strengthening-global-health-security-at-the-human-animal-interface (Accessed May 1, 2025).

[2] Di Bari C Venkateswaran N Fastl C Gabriël S Grace D Havelaar AH. The global burden of neglected zoonotic diseases: current state of evidence. One Health. (2023) 17:100595. doi: 10.1016/j.onehlt.2023.100595, PMID: 37545541 PMC10400928

[3] Thumbi SM Blumberg L le Roux K Salahuddin N Abela B. A call to accelerate an end to human rabies deaths. Lancet. (2022) 400:2261–4. doi: 10.1016/S0140-6736(22)02487-4, PMID: 36528379 PMC9754655

[4] Mpouam SE Mingoas JPK Mouiche MMM Feussom JMK Saegerman C. Critical systematic review of Zoonoses and transboundary animal diseases’ prioritization in Africa. Pathogens. (2021) 10:976. doi: 10.3390/pathogens10080976, PMID: 34451440 PMC8401284

[5] WOAH. Rabies control: A model for one health collaboration - WOAH. (2022). Available online at: https://www.woah.org/en/rabies-control-a-model-for-one-health-collaboration/ (Accessed November 17, 2024).

[6] Hampson K Coudeville L Lembo T Sambo M Kieffer A Attlan M. Estimating the global burden of endemic canine rabies. PLoS Negl Trop Dis. (2015) 9:e0003709. doi: 10.1371/journal.pntd.0003709, PMID: 25881058 PMC4400070

[7] WHO. Rabies. (2024) Available online at: https://www.who.int/health-topics/rabies#tab=tab_1 (Accessed July 28, 2024).

[8] Munyua P Bitek A Osoro E Pieracci EG Muema J Mwatondo A. Prioritization of zoonotic diseases in Kenya, 2015. PLoS One. (2016) 11:e0161576. doi: 10.1371/journal.pone.0161576, PMID: 27557120 PMC4996421

[9] Cleaveland S Fèvre EM Kaare M Coleman PG. Estimating human rabies mortality in the United Republic of Tanzania from dog bite injuries. Bull World Health Organ. (2002) 80:304–10. doi: 10.1590/S0042-96862002000400009 12075367 PMC2567765

[10] Ngugi JN Maza AK Omolo OJ Obonyo M. Epidemiology and surveillance of human animal-bite injuries and rabies post-exposure prophylaxis, in selected counties in Kenya, 2011-2016. BMC Public Health. (2018) 18. doi: 10.1186/s12889-018-5888-5, PMID: 30092769 PMC6085719

[11] ZDU Kenya. Rabies elimination strategy. (2014).

[12] Muinde P Bettridge JM Sousa FM Dürr S Dohoo IR Berezowski J. Who let the dogs out? Exploring the spatial ecology of free-roaming domestic dogs in western Kenya. Ecol Evol. (2021) 11:4218–31. doi: 10.1002/ece3.7317, PMID: 33976805 PMC8093722

[13] Knobel DL Cleaveland S Coleman PG Fèvre EM Meltzer MI Elizabeth M. Re-evaluating the burden of rabies in Africa and Asia. Bull World Health Organ. (2005) 83:360–8. doi: 10.1590/S0042-96862005000500012 PMID: 15976877 PMC2626230

[14] GARC, WHO, WOAH, and FAO. ZERO BY 30 the global strategic plan to end human deaths from dog-mediated rabies by 2030. (2018). Available online at: www.who.int

[15] Kitala P Mcdermott J Kyule M Gathuma J Perry B Wandeler A. Dog ecology and demography information to support the planning of rabies control in Machakos District, Kenya. Acta Trop. (2001) 78:217–30. doi: 10.1016/S0001-706X(01)00082-1, PMID: 11311185

[16] Wambura G Mwatondo A Muturi M Nasimiyu C Wentworth D Hampson K. Rabies vaccine and immunoglobulin supply and logistics: challenges and opportunities for rabies elimination in Kenya. Vaccine. (2019) 37:A28–34. doi: 10.1016/j.vaccine.2019.05.035, PMID: 31326251 PMC7612384

[17] Bitek AO Osoro E Munyua PM Nanyingi M Muthiani Y Kiambi S. A hundred years of rabies in Kenya and the strategy for eliminating dog-mediated rabies by 2030. AAS Open Res. (2018) 1:23. doi: 10.12688/aasopenres.12872.1 32259023 PMC7117960

[18] Odinga CO Thomas LF Wambugu E Ferguson AW Fèvre EM Gibson A. Integrated community-based reporting and field diagnostics for improved rabies surveillance in rural Laikipia, Kenya. Zoonoses Public Health. (2025) 72:194–9. doi: 10.1111/zph.13193, PMID: 39618401 PMC11772914

[19] MOH and WHO. IDSR standard case definitions for immediately reportable (notifiable) diseases. Kenya: Ministry of Health (2024).

[20] Chuchu VM Kitala PM Bichanga P Ksee D Muturi M Mwatondo A. Rabies elimination in rural Kenya: need for improved availability of human vaccines, awareness and knowledge on rabies and its management among healthcare workers. Front Pub Health. (2022) 10:769898. doi: 10.3389/fpubh.2022.769898, PMID: 35356016 PMC8960031

[21] Kenya DVS. Kenya animal disease surveillance manual. Kenya: WHO (2018).

[22] Rysava K Espineda J Silo EAV Carino S Aringo AM Bernales RP. One health surveillance for rabies: a case study of integrated bite case Management in Albay Province, Philippines. Front Trop Dis. (2022) 3:787524. doi: 10.3389/fitd.2022.787524, PMID: 40453584

[23] Kahariri S Thumbi SM Bett B Mureithi MW Nyaga N Ogendo A. The evolution of Kenya’s animal health surveillance system and its potential for efficient detection of zoonoses. Front Vet Sci. (2024) 11:1379907. doi: 10.3389/fvets.2024.1379907, PMID: 38966562 PMC11223174

[24] Kwoba EN Kitala P Ochieng L Otiang E Ndung’u R Wambura G. Dog health and demographic surveillance survey in Western Kenya: demography and management practices relevant for rabies transmission and control. AAS Open Res. (2019) 2:5. doi: 10.12688/aasopenres.12902.1

[25] Thumbi SM Muema J Mutono N Njuguna J Jost C Boyd E. The livestock for health study: a field trial on livestock interventions to prevent acute malnutrition among women and children in pastoralist communities in northern Kenya. Food Nutr Bull. (2023) 44:S119–23. doi: 10.1177/03795721231195427, PMID: 37850922

[26] Bunn AG. A dendrochronology program library in R (dplR). Dendrochronologia. (2008) 26:115–24. doi: 10.1016/j.dendro.2008.01.002

[27] van Niekerk J Rue H Bakka H Schenk O. New Frontiers in Bayesian modeling using the INLA package in R. J Stat Softw. (2021) 100:1–28. doi: 10.18637/jss.v100.i02

[28] Monje F Kadobera D Ndumu DB Bulage L Ario AR. Trends and spatial distribution of animal bites and vaccination status among victims and the animal population, Uganda: a veterinary surveillance system analysis, 2013–2017. PLoS Negl Trop Dis. (2021) 15:e0007944. doi: 10.1371/journal.pntd.0007944, PMID: 33872314 PMC8084341

[29] Broban A Tejiokem MC Tiembré I Druelles S L’Azou M. Bolstering human rabies surveillance in Africa is crucial to eliminating canine-mediated rabies. PLoS Negl Trop Dis. (2018) 12:e0006367. doi: 10.1371/journal.pntd.0006367, PMID: 30188896 PMC6126826

[30] Punyapornwithaya V Thanapongtharm W Jainonthee C Chinsorn P Sagarasaeranee O Salvador R. Time series analysis and forecasting of the number of canine rabies confirmed cases in Thailand based on national-level surveillance data. Front Vet Sci. (2023) 10:1294049. doi: 10.3389/fvets.2023.1294049, PMID: 38094496 PMC10716232

[31] Baker L Matthiopoulos J Müller T Freuling C Hampson K. Local rabies transmission and regional spatial coupling in European foxes. PLoS One. (2020) 15:e0220592. doi: 10.1371/journal.pone.0220592, PMID: 32469961 PMC7259497

[32] González-Roldán JF Undurraga EA Meltzer MI Atkins C Vargas-Pino F Gutiérrez-Cedillo V. Cost-effectiveness of the national dog rabies prevention and control program in Mexico, 1990–2015. PLoS Negl Trop Dis. (2021) 15:e0009130. doi: 10.1371/journal.pntd.0009130, PMID: 33661891 PMC7963054

[33] Karani M Muloi D Njenga G Thomas LF Fèvre EM. Kenya’s rabies battle: Unmasking the silent scourge. (2023). ILRI. Available online at: https://hdl.handle.net/10568/132349

[34] Thomas LF Rushton J Bukachi SA Falzon LC Howland O Fèvre EM. Cross-sectoral zoonotic disease surveillance in Western Kenya: identifying drivers and barriers within a resource constrained setting. Front Vet Sci. (2021):8. doi: 10.3389/fvets.2021.658454/full PMC8217437 34169106

[35] Swedberg C Mazeri S Mellanby RJ Hampson K Chng NR. Implementing a one health approach to rabies surveillance: lessons from integrated bite case management. Front Trop Dis. (2022) 3:829132. doi: 10.3389/fitd.2022.829132, PMID: 36945698 PMC7614337

[36] Ghosh S Hasan MN Nath ND Haider N Jones DH Islam MK. Rabies control in Bangladesh and prediction of human rabies cases by 2030: a one health approach. Lancet Regional Health Southeast Asia. (2024) 27:100452. doi: 10.1016/j.lansea.2024.100452, PMID: 39140082 PMC11321326

[37] Schrodt CA Dilius P Gibson AD Crowdis K Fénelon N Ross Y. Electronic application for rabies management improves surveillance, data quality, and investigator experience in Haiti. Front Vet Sci. (2023) 10:1052349. doi: 10.3389/fvets.2023.1052349, PMID: 37065250 PMC10103903

[38] Barroga TRM Basitan IS Lobete TM Bernales RP Gordoncillo MJN Lopez EL. Community awareness on rabies prevention and control in Bicol, Philippines: pre- and post-project implementation. Trop Med Infect Dis. (2018) 3:16. doi: 10.3390/tropicalmed3010016, PMID: 30274414 PMC6136611

[39] Dürr S Naïssengar S Mindekem R Diguimbye C Niezgoda M Kuzmin I. Rabies diagnosis for developing countries. PLoS Negl Trop Dis. (2008) 2:e206. doi: 10.1371/journal.pntd.0000206, PMID: 18365035 PMC2268742

[40] World Health Organization, WHO expert consultation on rabies. WHO expert consultation on rabies: Second report. 149. 24069724

