# Supplementary figures and images for: One health surveillance: linking human and animal rabies surveillance data in Kenya
Source: Front Public Health. 2025 Jun 16;13:1594162. doi: 10.3389/fpubh.2025.1594162 (PMC12206715; doi:10.3389/fpubh.2025.1594162)

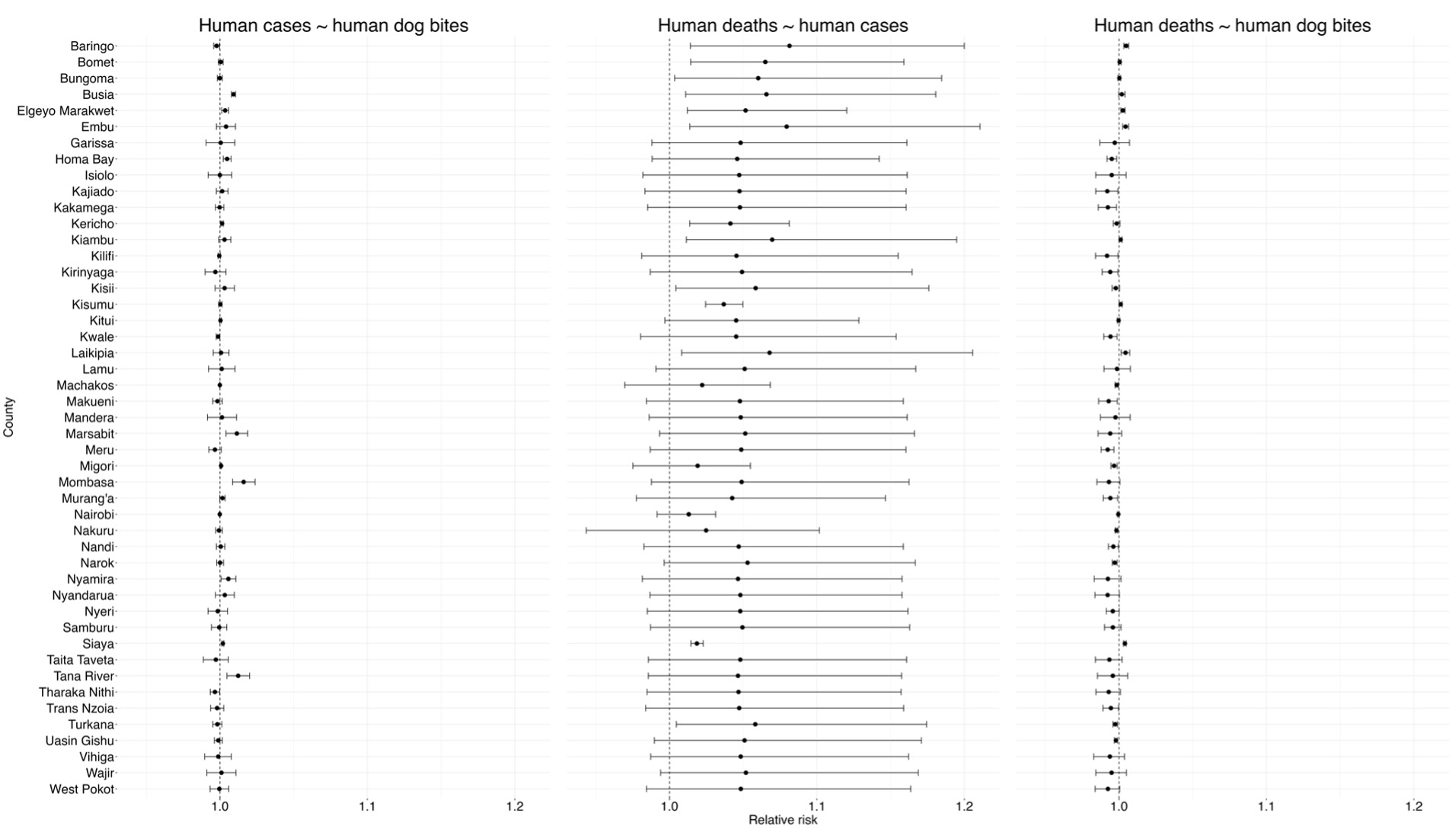

Supplement: Supplementary file 1 [file Image_1.JPEG]

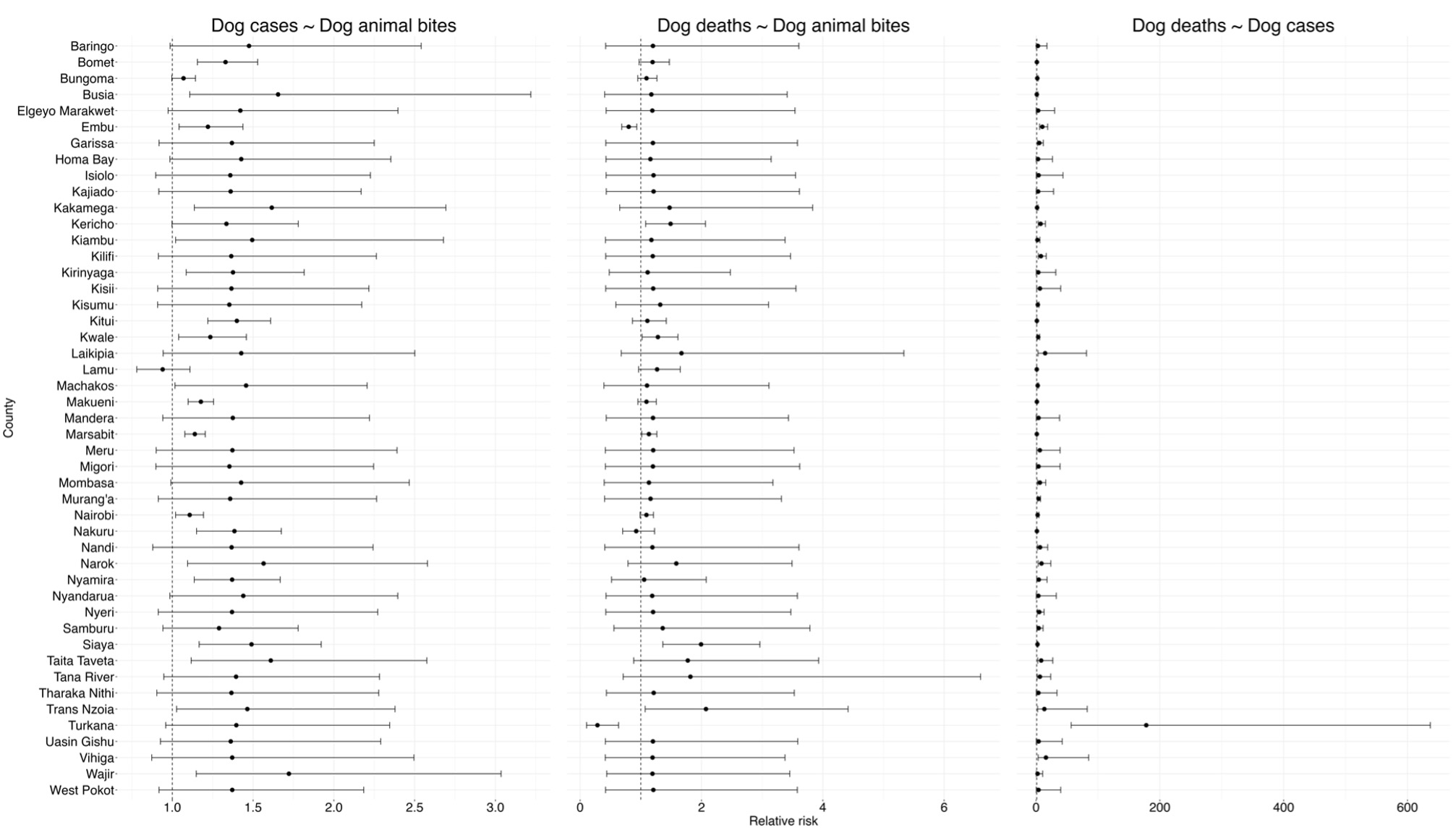

Supplement: Supplementary file 2 [file Image_2.JPEG]
